# A Rare and Challenging Presentation of an Inflammatory Myofibroblastic Tumour: A Case Report

**DOI:** 10.1002/rcr2.70232

**Published:** 2025-06-09

**Authors:** Rohit Shirgaonkar, Srivatsa Lokeshwaran, Susmita Rakshit

**Affiliations:** ^1^ Department of Interventional Pulmonology and Lung Transplant Aster Hospital Whitefield Bangalore India; ^2^ Department of Pathology Aster Hospital Whitefield Bangalore India

**Keywords:** cryobiopsy, debulking, Inflammatory Myofibroblastic tumour, rigid bronchoscopy, stent

## Abstract

Inflammatory Myofibroblastic Tumours (IMTs) are rare pulmonary neoplasms, accounting for less than 1% of lung tumours. Patients present with non‐specific symptoms and diverse radiological findings. A 32‐year‐old female presented with progressively worsening cough, haemoptysis, low‐grade fever and increasing shortness of breath. Chest X‐ray revealed a total right lung collapse. Further imaging revealed a mass obstructing the right mainstem bronchus. A staged debulking procedure using rigid bronchoscopy was performed, followed by the insertion of a silicone stent to maintain airway patency. Endobronchial growth turned out to be an Inflammatory Myofibroblastic tumour. The patient showed significant improvement in respiratory function and lung re‐expansion. Adjuvant therapy with ALK inhibitor was initiated. IMTs, while benign histologically, may recur or metastasise, requiring long‐term monitoring. Bronchoscopic interventions offer effective treatment for localised disease, while surgery is preferred for extensive tumours. Early diagnosis, genetic profiling and a multidisciplinary approach are key for optimal outcomes.

## Graphical abstract

Inflammatory Myofibroblastic Tumours (IMTs) are rare pulmonary neoplasms, accounting for less than 1% of lung tumours. Patients present with non‐specific symptoms and diverse radiological findings. We present the diagnostic work‐up and treatment for a rare and challenging case of IMTs in a 32‐year‐old female. Early diagnosis, genetic profiling and a multidisciplinary approach are key for optimal outcomes.

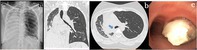

## 1 Introduction

IMTs are rare pulmonary tumours with an incidence rate of < 1%. The patient presents with non‐specific symptoms and diverse radiological findings. Histologically, these tumours exhibit spindle cell proliferation with lymphocyte infiltrate. Immunological staining is positive for ALK in 50%–60% of tumours. Complete resection is the primary treatment option for invasive tumours. Chemotherapy is useful in cases of multifocal, invasive lesions or cases of local recurrence.

## 2 Case Report

**FIGURE 1:**
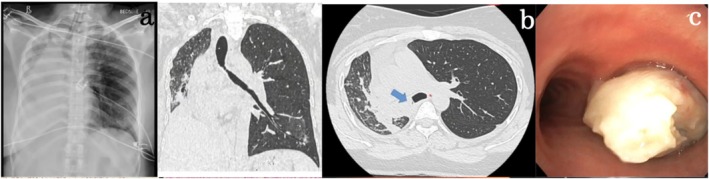
Chest Radiograph showing complete opacification of the right hemithorax (a). The lesion measuring approx. 2.3 cm × 1.3 cm in maximum dimensions with cephalon‐caudal extent of 3.2 cm in the right main bronchus. Superiorly the lesion is seen to involve upto the carina. Inferiorly the lesion is seen to extend into bronchus intermedius causing collapse of right middle, lower lobes (b). Smooth, vascular and friable growth was seen at the opening of the right mainstem bronchus; growth was seen to encroach into all the major segments of the right upper, middle and lower lobes (c).

**FIGURE 2:**
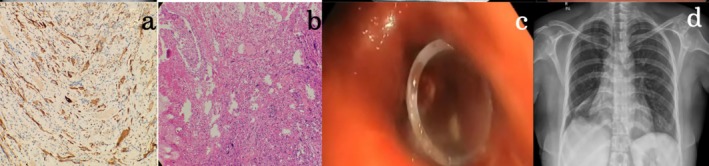
ALK, positive (diffuse, strong cytoplasmic expression in plump to spindle‐shaped cells) on IHC (a). Haematoxylin–eosin staining (20×) showing singly scattered plump to spindle‐shaped atypical cells with enlarged vesicular nuclei. Very prominent inclusion like nucleoli with moderate cytoplasm (b). A silicone stent measuring 10 mm × 3 mm is placed in the right lower lobe bronchus (c). A chest radiograph post‐procedure shows complete expansion of the right lung (d).

A 32‐year‐old female presented with a progressively worsening cough, haemoptysis, intermittent low‐grade fever and increasing shortness of breath for the last 6 months. She had no significant past medical history. Upon admission, her blood pressure was 120/78 mmHg, heart rate was 86/min, respiratory rate was 20/min, and oxygen saturation was 97% on room air. Physical examination did, however, reveal absent breath sounds over the right hemithorax. The initial laboratory data revealed haemoglobin of 12.5 g/dL (range: 12–16 g/dL), platelet count of 220,000/μL (range: 150,000–450,000/μL), white blood cell count of 13,000/μL (range: 4000–10,000/μL), and C‐reactive protein level of 18 mg/L (normal: < 5 mg/L). Her liver and kidney function tests were within normal limits. A chest X‐ray showed a complete opacification of the right lung (Figure 1a). A contrast‐enhanced CT thorax was conducted to evaluate the possible underlying reason for collapse, which showed a soft tissue mass completely occluding the right mainstem bronchi, causing post‐obstructive collapse of the entire right lung (Figure 1b). A check bronchoscopy revealed a friable and vascular endobronchial mass close to the carina that entirely occluded the right mainstem bronchi (Figure 1c). As the growth was in close proximity to the carina with a lack of decent margin for surgical resection and creating a bronchial stump, surgery was deferred. Biopsy exhibited classic features of spindle cell proliferation with ALK positivity on IHC, confirming the mass as Inflammatory Myofibroblastic (IMT) (Figure 2a,b). A staged bronchoscopic debulking procedure was planned. In the first setting, cryo‐debulking was done for 50% of the tumour mass. Tamponade with a Fogarty balloon and argon plasma coagulation (APC) were used to control the bleeding successfully. In the second session, additional debulking was done to remove the remnant tumour mass. The remnant tumour tissue was removed with coring using a large barrel bronchoscope, followed by a small barrel rigid bronchoscope. A silicone stent measuring 10 mm × 3 mm was placed in the right lower lobe bronchus due to its partial collapsibility (Figure 2c). A repeat chest X‐ray showed significant re‐expansion of the right lung (Figure 2d). The patient remained stable and demonstrated significant clinical improvement in respiratory function. A multidisciplinary team, including medical oncology, was consulted for further management. Given the ALK‐positive nature of the tumour, the patient was started on crizotinib, an ALK inhibitor. The patient will undergo PET‐CT imaging every 6 months to monitor for any signs of tumour recurrence. This surveillance plan aims to enable timely intervention in case of recurrence, with a multidisciplinary approach involving surgical re‐evaluation. After a month, the patient is symptomatically better. Follow‐up bronchoscopy has revealed no recurrence and that the stent is in place.

## 3 Discussion

Inflammatory Myofibroblastic Tumours (IMTs) account for approximately 0.04% to 1% of all lung tumours. These tumours exhibit no distinct gender or ethnic predilection, although they are reported in individuals under the age of 40 years. Patients have symptoms such as cough, dyspnea, fever, fatigue and hemoptysis [1]. Post‐obstructive collapse can also be a finding if the tumour presents as an endobronchial lesion. Although IMTs are considered histologically benign, metastasis can develop in 2%–5% of diagnosed cases due to angioinvasion, while local recurrence may occur in up to 25% of resected tumours. Approximately 50%–70% of these tumours test positive for genes related to the mitogen‐activated protein kinase (MAPK) pathway, particularly the ALK (Anaplastic Lymphoma Kinase), ROS1, PDGFR (Platelet‐Derived Growth Factor Receptor), and RET genes. Molecular profiling is essential to guide therapy and improve outcomes in these cases. Among ALK‐negative tumours, ROS1 fusions are the most commonly identified and have shown significant responses to the tyrosine kinase inhibitor crizotinib. Other alternative kinase fusions, such as RET, NTRK and PDGFRβ, many of which are targetable with FDA‐approved therapies [2]. In patients with localised disease and no signs of extensive endobronchial invasion, bronchoscopy‐aided debulking remains a promising option. Bronchoscopic techniques such as cryotherapy, neodymium laser therapy, photodynamic therapy and argon plasma coagulation (APC) have all been reported as useful options for treating endobronchial IMTs. Cryotherapy is useful both for debulking and local control of the tumour when applied at the lesion's base [3]. Additionally, radial endobronchial ultrasound (EBUS) can also be employed to evaluate the extent of tumour invasion within the bronchial wall. The effectiveness of these interventional techniques is highly dependent on the location and extent of the tumour [4]. In cases where the tumour extends beyond the bronchial cartilage or involves the lung parenchyma, surgery remains the treatment of choice due to the higher likelihood of recurrence. The use of ALK inhibitors, such as crizotinib, is particularly promising for tumours that harbour ALK gene rearrangements [5]. This disparity highlights the critical role of genetic testing in tailoring treatment plans to maximise efficacy. Thus, the diagnosis and treatment of IMT have evolved with advances in both interventional techniques and molecular understanding. While surgery remains the cornerstone of treatment, especially for tumours that invade beyond the bronchial cartilage, interventional bronchoscopic methods offer a minimally invasive option for patients with localised disease.

## Author Contributions

**Rohit Shirgaonkar:** writing – original draft. **Srivatsa Lokeshwaran:** writing, review and editing. **Susmita Rakshit:** pathology slides and discussion.

## Ethics Statement

The authors declare that appropriate written informed consent was obtained for the publication of this manuscript and accompanying images. Ethical approval was not required for this case report.

## Conflicts of Interest

The authors declare no conflicts of interest.

## Data Availability

The data that support the findings of this study are available on request from the corresponding author. The data are not publicly available due to privacy or ethical restrictions.

## References

[1] H. Sakurai , “Inflammatory Myofibroblastic Tumor of the Lung,” European Journal of Cardio‐Thoracic Surgery 25, no. 2 (2004): 155–159. 14747105 10.1016/s1010-7940(03)00678-x

[2] S. A. Debonis , A. Bongiovanni , F. Pieri , et al., “ALK‐Negative Lung Inflammatory Myofibroblastic Tumor in a Young Adult,” Medicine (Baltimore) 100, no. 20 (2021): e25972. 34011083 10.1097/MD.0000000000025972 PMC8137108

[3] N. S. Bham and J. D. Schwartz , “Endobronchial Inflammatory Myoblastic Tumor (IMT)—A Sleeve to Solve the Issue,” Annals of Thoracic Surgery Short Reports 3, no. 1 (2024): S2772993124003577. 10.1016/j.atssr.2024.08.009 PMC11910756 40098826

[4] A. Iyer , T. Radonic , L. C. Heukamp , E. Thunnissen , and J. M. A. Daniels , “Inflammatory Myofibroblastic Tumour of the Central Airways: Treatment and Molecular Analysis,” ERJ Open Research 7, no. 1 (2021): 00151‐2020. 33778057 10.1183/23120541.00151-2020 PMC7983254

[5] C. K. Chen , C. I. Jan , J. S. Tsai , et al., “Inflammatory Myofibroblastic Tumor of the Lung‐ a Case Report,” Journal of Cardiothoracic Surgery 5, no. 1 (2010): 55. 20646317 10.1186/1749-8090-5-55 PMC2915987

